# Increased genetic divergence between two closely related fir species in areas of range overlap

**DOI:** 10.1002/ece3.1007

**Published:** 2014-03-03

**Authors:** Jing Wang, Richard J Abbott, Pär K Ingvarsson, Jianquan Liu

**Affiliations:** 1 Key Laboratory of Bio-Source and Environmental Conservation, School of Life Science, Sichuan University6100061, Chengdu, Sichuan, China; 2 Umeå Plant Science Centre, Department of Ecology and Environmental Science, Umeå UniversitySE-90187, Umeå, Sweden; 3 School of Biology, Mitchell Building, University of St AndrewsSt Andrews, Fife KY16 9TH, UK

**Keywords:** *Abies chensiensis*, *Abies fargesii*, allopatry, genetic divergence, natural selection, parapatry

## Abstract

Because of introgressive hybridization, closely related species can be more similar to each other in areas of range overlap (parapatry or sympatry) than in areas where they are geographically isolated from each other (allopatry). Here, we report the reverse situation based on nuclear genetic divergence between two fir species, *Abies chensiensis* and *Abies fargesii*, in China, at sites where they are parapatric relative to where they are allopatric. We examined genetic divergence across 126 amplified fragment length polymorphism (AFLP) markers in a set of 172 individuals sampled from both allopatric and parapatric populations of the two species. Our analyses demonstrated that AFLP divergence was much greater between the species when comparisons were made between parapatric populations than between allopatric populations. We suggest that selection in parapatry may have largely contributed to this increased divergence.

## Introduction

Introgression commonly occurs between closely related species in areas where their distributions overlap (Anderson and Hubricht 1938; Rieseberg and Wendel 1993; Sullivan et al. 2004; Mehner et al. 2010). This can lead to two species being genetically more similar in areas of range overlap (parapatry or sympatry) than in areas where they are geographically isolated from each other (allopatry) (Palme et al. 2004; Behm et al. 2010; McKinnon et al. 2010). Alternatively, selection can act to minimize resource competition or reproductive interference between closely related species in parapatry (or sympatry), thereby promoting exaggerated interspecific divergence and thus enabling them to coexist (Pfennig and Pfennig 2010). Accordingly, a distinct pattern of increased interspecific differentiation between closely related species in parapatry (or sympatry) compared with that in allopatry has been reported for ecological and reproductive traits in a number of animals and plants (Levin 1978; Sætre et al. 1997; Grant and Grant 2006; Niet et al. 2006; Kay and Schemske 2008; Smith and Rausher 2008; Urbanelli and Porretta 2008; Kirschel et al. 2009; Grossenbacher and Whittall 2011). This process of divergence of characters between species in response to selection is usually termed “character displacement” (Brown and Wilson 1956; Pfennig and Pfennig 2009).

**Figure 1:**
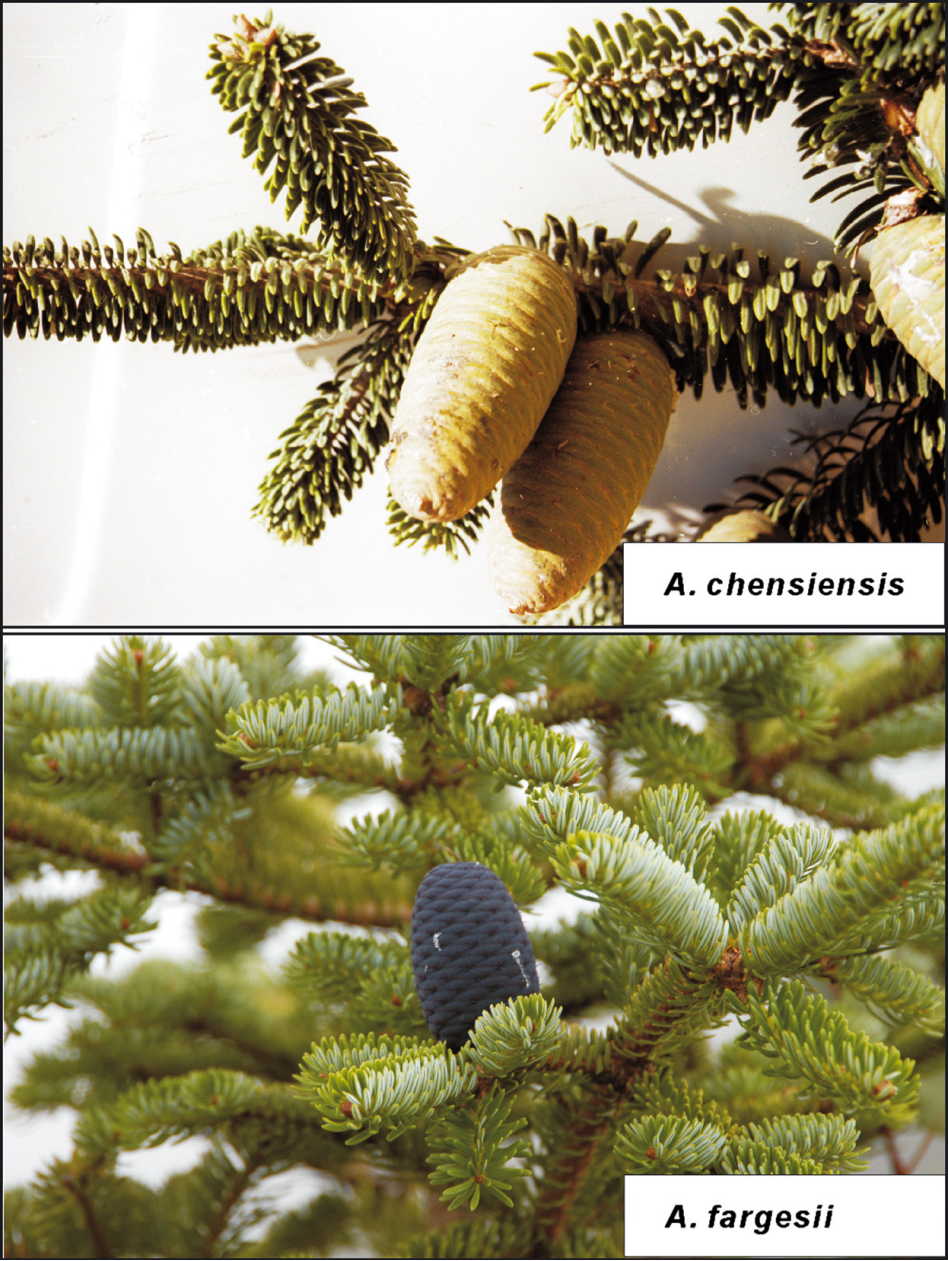
The photo shows phenotypic differences between *Abies chensiensis* and *Abies fargesii*.

Although empirical and theoretical work on character displacement has illuminated the role of selection in promoting divergent evolution and triggering speciation (Pfennig and Pfennig 2009; Hoskin and Higgie 2010; Hopkins and Rausher 2011), relatively little work has explored the extent to which selection could potentially reduce gene flow and contribute further to genetic divergence across the genome (Hoskin et al. 2005; Hopkins et al. 2012; Nosil et al. 2012). The analysis of genomic patterns of divergence between incipient species experiencing secondary contact represents a powerful approach to address these questions. In this study, we focus on two fir species, *Abies chensiensis* and *Abies fargesii* from central China. Most populations of these two species are allopatric, but in some areas the species co-occur (Fu et al. 1999). In allopatry, both species usually grow at elevations from 1500 to 3000 m, but in areas of parapatry *A. chensiensis* is more abundant at lower elevations, whereas *A. fargesii* tends to occupy sites at higher altitudes (Guan 1982; Zhang 2001). Morphologically, the two species are distinguished by the color of branchlets and seed cones, the morphology of bracts, and the shape and color of leaf needles (Fu et al. 1999) (Fig. 1). Accordingly, *A. chensiensis* and *A. fargesii* have been grouped into two different sections on the basis of the most widely recognized classifications (Liu 1971; Farjon and Rushforth 1989). Nevertheless, a lack of genetic differences between them for both chloroplast (cp) and mitochondrial (mt) DNA sequences indicates that the two fir species are in fact closely related and might represent an incipient stage of speciation, with insufficient divergence time having occurred for complete lineage sorting of ancestral polymorphisms (Wang et al. 2011).

Based on the distribution of cpDNA and mtDNA diversity within and between both species, it has been suggested that parapatric populations likely represent secondary contact zones between the species, which formed after the penultimate glacial period (Wang et al. 2011). Because introgression frequently occurs in conifers, especially in secondary contact zones (Liepelt et al. 2002; Petit et al. 2005; Semerikova et al. 2011), we might expect that introgression will have reduced genetic divergence between these two species in parapatric compared with allopatric sites. Alternatively, speciation in parapatry may have been facilitated or even finalized by the evolution of reproductive barriers through the process of character displacement (Pfennig and Pfennig 2010; Abbott et al. 2013; Hopkins 2013). If this is correct, loci under divergent selection (plus neutral sites tightly linked to them) may exhibit enhanced genetic divergence in parapatry relative to other parts of the genome subject to weak or no selection, and where lineage sorting is incomplete and/or where the homogenizing effects of gene flow are more extreme (Nosil et al. 2009; Hopkins et al. 2012). Divergent selection may also reduce gene flow globally across the genome, facilitating the accumulation of genome-wide divergence between parapatric populations, although the degree of divergence might still be substantially heterogeneous across the genome depending on the distribution of selected sites (Nosil et al. 2008; Feder et al. 2012; Flaxman et al. 2013). The goals of this study were (1) to determine whether there is increased or reduced genetic differentiation between parapatric populations compared with allopatric populations of these two recently diverged fir species; (2) to explore how genetic differentiation is distributed throughout the genome and whether regions of divergence are concentrated in just a few ‘genomic islands’ or is widespread across the genome.

## Materials and Methods

### Sampling

**Figure 2:**
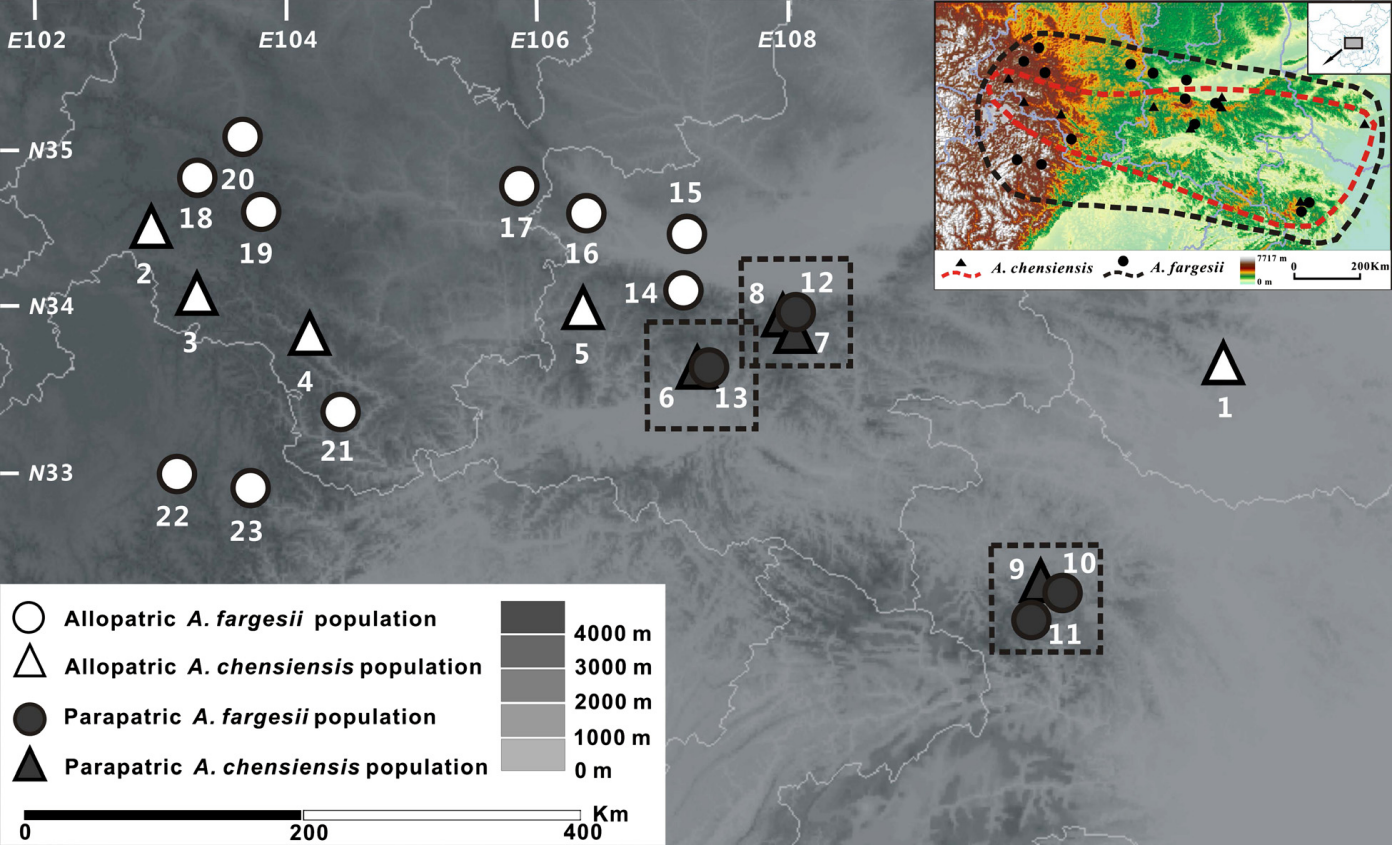
Sampling localities and distributions of *Abies chensiensis* (marked by black solid triangle and red dashed line) and *Abies fargesii* (marked by black solid circle and black dashed line) in central China (in the upper right corner). Sampled populations (details in Table (1).) in this study include both allopatric and parapatric populations of *Abies chensiensis* (marked by white and black triangles) and *A. fargesii* (marked by white and black circles). Parapatric areas are shown in three independent dashed squares.

We sampled indigenous, phenotypically pure populations of *A. chensiensis* and *A. fargesii* from almost the entire range of the two species in China. In total, five populations of *A. chensiensis* and 10 populations of *A. fargesii* were sampled from allopatric sites, while four populations of each species were sampled from sites where they were parapatric (Table 1; Fig. 2). We specified populations as parapatric if the two species grew together in the same valley or on the same mountain with *A. fargesii* occurring above 2200 m, and *A. chensiensis* at lower elevations. Fresh needles of individuals, spaced at least 100 m apart, were collected from each population and dried in silica gel. Representative vouchers were made from each population and deposited in the Lanzhou University herbarium.

**Table 1:** Description of allopatric and parapatric populations sampled in *Abies chensiensis* and *Abies fargesii*.

Population	Location	Region	Latitude(N)	Longitude(E)	Altitude (m)	*N*	% polymorphic loci	*H*_e_
*A. chensiensis*								
1	Neixiangxian HN	Allopatry	33°02.415′	111°50.578′	2516	3	100.0	0.229
2	Yiwaxiang GS	Allopatry	34°11.652′	103°11.810′	2776	14	42.1	0.210
3	Lazikou GS	Allopatry	33°48.285′	103°41.583′	2441	8	95.2	0.199
4	Zhouqu GS	Allopatry	33°33.467′	104°20.738′	2058	6	94.4	0.246
5	Lueyang SX	Allopatry	33°27.842′	106°27.638′	1380	5	94.4	0.254
6	Foping SX	Parapatry	33°15.075′	107°59.491′	1986	7	93.7	0.281
7	Ningshanxian SX	Parapatry	33°31.455′	108°21.111′	1366	8	91.3	0.213
8	Ningshanxian SX	Parapatry	33°47.172′	108°20.227′	2012	5	88.1	0.246
9	Shennongjia HB	Parapatry	31°48.037′	110°30.101′	1809	12	93.7	0.284
Subtotal						68		
*A. fargesii*								
10	Shennongjia HB	Parapatry	31°42.347′	110°39.157′	2256	4	83.3	0.199
11	Shennongjia HB	Parapatry	31°27.108′	110°17.011′	2650	16	91.3	0.242
12	Ningshanxian SX	Parapatry	33°47.172′	108°20.227′	2573	5	84.9	0.196
13	Foping SX	Parapatry	33°15.075′	107°59.491′	2420	13	32.5	0.169
14	Zhouzhi SX	Allopatry	33°51.058′	107°50.355′	2189	4	100.0	0.303
15	Meixian SX	Allopatry	34°09.546′	107°50.383′	2680	7	94.4	0.204
16	Xinjiashan SX	Allopatry	34°16.219′	106°31.562′	2097	10	95.2	0.223
17	Huoyanshan GS	Allopatry	34°22.546′	106°14.201′	2563	12	93.7	0.226
18	Lintan GS	Allopatry	34°54.407′	103°41.351′	2945	5	92.1	0.217
19	Diebu GS	Allopatry	34°08.232′	103°53.022′	2600	8	93.7	0.234
20	Lianhuashan GS	Allopatry	34°56.453′	103°45.470′	3196	7	90.5	0.233
21	Wenxian GS	Allopatry	32°57.522′	104°38.076′	2357	5	89.7	0.242
22	Chuanzhusi SC	Allopatry	32°46.407′	103°37.220′	2688	5	92.9	0.292
23	Huanglong SC	Allopatry	32°45.011′	103°49.133′	3291	3	100.0	0.287
Subtotal						104		
Total						172		

N, number of individuals analyzed; % polymorphic loci, the percentage of loci that are polymorphic out of the total 126 loci; *H*_e_, mean expected heterozygosity.

HN, Henan; GS, Gansu; SX, Shanxi; HB, Hebei; SC, Sichuan.

### DNA extraction and AFLP genotyping

Genomic DNA was extracted from approximately 20 mg of silica gel-dried, needle material per sample according to a cetytrimethyl ammonium bromide (CTAB) procedure (Doyle and Doyle 1987). Amplified fragment length polymorphism profiles were generated using a protocol modified slightly from Vos et al. (1995). An initial screening of selective primers was performed on six individuals from six populations, using 20 primer combinations with three selective nucleotides. We selected three *Eco*RI/*Mse*I primer pairs producing the most repeatable and unambiguously scorable profiles. Fluorescence-labeled fragments were separated on a CEQ 8000 capillary sequencer (Beckman Coulter), with an internal size standard. All loci between 100 and 500 bp were then visually inspected in all individuals. Only unambiguously scorable loci and individuals were included in the analysis, and peaks found in <3% of individuals were excluded. In total, 172 individuals were scored for 126 AFLP markers, with all scoring being performed blind to population of origin. To ensure high repeatability of analyzed AFLP loci, we ran a subset of 30 individuals twice from the preselective amplification step. The average per-locus genotyping error rate for the AFLP data, measured as recommended by Bonin et al. (2004), was 2.6%.

### Genetic diversity and population structure

To assess genetic diversity in each population, the percentage of polymorphic fragments and Nei's expected heterozygosity (*H*_e_) were determined. We also estimated total diversity (*H*_T_), within-population diversity (*H*_S_), between-population diversity (*H*_B_), and population differentiation (*F*_ST_) for allopatric and parapatric populations of each species, taken separately and in combination, using the method of Lynch and Milligan (1994) as implemented in the program AFLP-SURV version 1.0 (Vekemans et al. 2002). Because the inbreeding coefficient (*f*) cannot be calculated directly due to the dominant nature of AFLPs, we employed an alternative Bayesian approach developed by Holsinger et al. (2002) to estimate an *F*_ST_ analogue (designated by *θ*^B^) for dominant markers, which accounts for uncertainty in *f* and simultaneously provides accurate and reliable estimates of genetic differentiation (Holsinger and Wallace 2004; Holsinger and Lewis 2007). Using the program HICKORY version 1.1 (Holsinger and Lewis 2007), we ran three models separately: (1) full model (both *θ*^B^ and *f* are unknown); (2) *f *=* *0 model (*θ*^B^ is unknown and no inbreeding occurs); (3) *f*-free model (*f* was not estimated but was chosen at random from the prior distribution). The posterior distribution of *θ*^B^ was estimated through Markov Chain Monte Carlo (MCMC) methods by HICKORY v1.1, with a burn-in of 5000 iterations and a sampling run of 25,000 iterations from which every fifth sample was retained for posterior calculations. The deviance information criterion (DIC) was subsequently used to determine the model showing the best fit to the data (Holsinger and Wallace 2004). To reveal differentiation patterns, we calculated pairwise *F*_ST_ and *θ*^B^ values between a priori defined groups based on various combinations of species (*A. chensiensis* and *A. fargesii*) and population type (allopatric and parapatric) using AFLP-SURV 1.0 and HICKORY 1.1. The significance of *F*_ST_ values was calculated based on comparison with values obtained from 10,000 randomly permuted individuals among the populations and/or between groups. We then constructed a neighbor-joining (NJ) tree with the program NEIGHBOR incorporated in the software package PHYLIP version 3.6 (Felsenstein 2004). Bootstrap support for internal nodes was estimated with a 10,000 distance matrix of replicates generated by AFLP-SURV 1.1, and a consensus tree was generated with CONSENSE in PHYLIP.

To visualize the relative genetic relationships between individuals and populations of *A. chensiensis* and *A. fargesii*, we conducted a principal coordinate analysis (PCoA) on a genetic distance matrix generated from the binary presence–absence matrix as implemented in GENALEX 6.2 (Peakall and Smouse 2006). Principal coordinate model clustering (PCO-MC), which couples PCoA with a clustering procedure, was used to further determine significant population structure in the AFLP dataset following Reeves and Richards (2009) and http://lamar.colostate.edu/∼reevesp/PCOMC/PCOMC.html. Rather than only visualizing the first two or three principal coordinate axes, this method simultaneously takes into consideration information for all axes of a PCoA and offers an object way to determine whether clusters found in the PCoA are significant using NTSYS and the MODECLUS procedure in SAS 9.1. In addition, hierarchical partitioning of genetic diversity was estimated by performing analyses of molecular variance (AMOVA) (Excoffier et al. 1992) on populations grouped according to population type (allopatric and parapatric) within each species, and/or according to species (*A. chensiensis* and *A. fargesii*) as implemented for dominant markers in GENALEX 6.2 (Peakall and Smouse 2006), with significance tested by a nonparametric permutation procedure with 9999 permutations.

We checked whether genetic structure between parapatric and allopatric populations of *A. chensiensis* and *A. fargesii* was correlated with geographical distance, according to a pattern of isolation by distance (IBD). Pairwise genetic distance between populations was estimated using the Slatkin's linearized *F*_ST_ values (*F*_ST_/1−*F*_ST_) in ARLEQUIN 3.5 (Rousset 1997; Excoffier and Lischer 2010). Then, a Mantel test was performed on pairwise values of linearized *F*_ST_ and log-transformed geographical distances. The significance of these correlations was evaluated with 1000 permutations using the VEGAN package in R (R Development Core Team 2013). To compare the overall distribution of genetic differentiation across the genome between parapatric and allopatric populations of these two species, we used the Bayesian method of Foll and Gaggiotti (2008) to calculate locus-specific estimates of *F*_ST_, which allows for the estimation of both population-specific effects (*β*_j_) and locus-specific effects (*α*_i_) on genetic differentiation and has been shown to be robust to complex demographic models.

## Results

**Figure 3:**
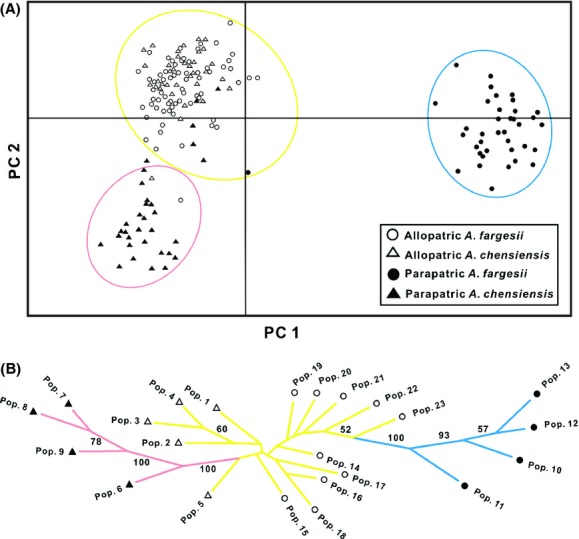
(A) Principal coordinate analysis of AFLP marker variation among 172 individuals in *Abies chensiensis* and *Abies fargesii*. The variance explained by PC1 and PC2 is 53.4% and 19.4%, respectively. The three clusters that were found to be significant in the PCO-MC analysis are circled by different color. (B) Unrooted neighbor-joining tree based on Nei's genetic distance between populations of *Abies chensiensis* and *Abies fargesii*. Populations are labeled as in Fig. 1, and branches are color-coded as in (A). Bootstrap values over 50 are shown next to the corresponding branches.

Of a total of 126 AFLP loci surveyed, 101 (80.2%) were polymorphic. Values of average gene diversity (*H*_e_) per population ranged between 0.199 and 0.284 for *A. chensiensis*, and between 0.169 and 0.303 for *A. fargesii* (Table 1). In each species, average within-population diversity (*H*_S_) was much higher than average between-population diversity (*H*_B_) for both allopatric and parapatric populations whether considered separately or together (Table 2). For all estimates of *θ*^B^, the full model showed the best fit to the data, having the lowest DIC value (data not shown), and thus, we only present the *θ*^B^ values from the full model. Population differentiation (*F*_ST_ and *θ*^B^) between parapatric or allopatric populations treated separately in both species was much lower than values of *F*_ST_ and *θ*^B^ obtained when all populations within species were considered together (Table 2). Estimates of genetic differentiation between *A. chensiensis* and *A. fargesii* in terms of *F*_ST_ and *θ*^B^ were much lower when allopatric populations were compared (*F*_ST_ = 0.0455, *θ*^B^ = 0.0533) than when comparisons were made only between parapatric populations (*F*_ST_ = 0.4053, *θ*^B^ = 0.4742) (Table 3). It was also the case that allopatric populations of *A*. *fargesii* were more similar to parapatric populations of *A*. *chensiensis* (*F*_ST_ = 0.1323, *θ*^B^ = 0.1904), than were allopatric populations of *A*. *chensiensis* to parapatric populations of *A*. *fargesii* (*F*_ST_ = 0.4040, *θ*^B^ = 0.4741). There was greater divergence between allopatric and parapatric populations in *A. fargesii* (*F*_ST_ = 0.3685, *θ*^B^ = 0.4411) than within *A*. *chensiensis* (*F*_ST_ = 0.1559, *θ*^B^ = 0.2178) (Table 3). A neighbor-joining tree constructed with Nei's genetic distances calculated for the entire AFLP dataset revealed that relationships between allopatric populations of both species were generally poorly resolved (bootstrap value <50) (Fig. 3B). By contrast, populations in parapatry were clearly structured into two distinct and highly supported clades (bootstrap value = 100) corresponding to the two species (Fig. 3B).

**Table 2:** Genetic diversity statistics for *Abies chensiensis*, *Abies fargesii*, and for allopatric and parapatric populations within each species.

Species	*H*_T_	*H*_S_	*H*_B_	*F*_ST_	*θ*^B^
*A. chensiensis*					
Allopatric populations	0.2297	0.2278	0.0020	0.0084NS	0.0310 (0.0076–0.0633)
Parapatric populations	0.2716	0.2560	0.0156	0.0573*	0.1009 (0.0641–0.1459)
Total	0.2705	0.2403	0.0302	0.1112*	0.1688 (0.1363–0.2036)
*A. fargesii*					
Allopatric populations	0.2563	0.2461	0.0102	0.0398*	0.0671 (0.0428–0.0957)
Parapatric populations	0.2141	0.2016	0.0125	0.0580*	0.0609 (0.0294–0.1008)
Total	0.2986	0.2334	0.0653	0.2197*	0.2992 (0.2670–0.3325)

*H*_T,_ total population diversity; *H*_S_, average within-population diversity; *H*_B_, average between-population diversity; *F*_ST_ and *θ*^B^, the population differentiation (*θ*^B^ with 95% credibility intervals in parentheses); NS, not significant.

* Significant at *P *<* *0.001.

**Table 3:** Matrices of pairwise *F*_ST_ (below diagonal) and *θ*^B^ (above diagonal) for comparisons of allopatric and parapatric populations between and within species.

		Allopatric		Parapatric	
Populations	Species	*A. chensiensis*	*A. fargesii*	*A. chensiensis*	*A. fargesii*
Total loci					
Allopatric	*A. chensiensis*	–	0.0533 (0.0319–0.0819)	0.2178 (0.1509–0.2996)	0.4741 (0.4043–0.5395)
	*A. fargesii*	0.0455*	–	0.1904 (0.1339–0.2632)	0.4411 (0.3658–0.5119)
Parapatric	*A. chensiensis*	0.1559*	0.1323*	–	0.4742 (0.4053–0.5354)
	*A. fargesii*	0.4040*	0.3685*	0.4053*	–

* Significant at *P *<* *0.001. *θ*^B^ with 95% credible intervals in parentheses following estimates.

A plot of individual scores against the first two principal coordinates extracted from the PCoA and accounting for 53.4% and 19.4% of the variance, respectively, divided the dataset into three significantly distinct clusters as identified by PCO-MC analysis (circled in Fig. 3A). The largest of these clusters mainly comprised allopatric individuals of the two species, while the second cluster was mainly composed of parapatric individuals of *A*. *chensiensis*, and the third cluster consisted of only parapatric *A*. *fargesii* individuals. In general, these patterns of divergence were confirmed by the results of AMOVA, which showed that while only 5% of total variation was accounted for by interspecific differences between allopatric populations, 52% of total variation was due to differences between species when parapatric populations were compared (Table 4). Furthermore, 25% and 48% of total variance was partitioned between allopatric and parapatric populations within *A*. *chensiensis* and *A*. *fargesii*, respectively. The percentage of genetic diversity measured among populations within population types (allopatric or parapatric) was very low in all comparisons (Table 4).

**Table 4:** Analysis of molecular variance (AMOVA) for AFLP variation within and between *Abies chensiensis* and *Abies fargesii* when individuals were grouped according to population type, that is, allopatric or parapatric.

Species/Regions	Source of variation	df	SS	Est. variance	% Variance	Value	*P* value
*A. chensiensis*	Among population type (allopatric vs. parapatric)	1	119.91	3.08	25	Φ_RT_ = 0.246	0.001
	Among populations within population type	7	98.44	0.75	6	Φ_PR_ = 0.079	0.001
	Within populations	59	514.35	8.72	69	Φ_PT_ = 0.305	0.001
	Total	67	732.71	12.55			
*A. fargesii*	Among population type (allopatric vs. parapatric)	1	428.02	8.52	48	Φ_RT_ = 0.478	0.001
	Among populations within population type	12	168.84	0.79	4	Φ_PR_ = 0.085	0.001
	Within populations	90	766.26	8.51	48	Φ_PT_ = 0.523	0.001
	Total	103	1363.13	17.83			
Allopatric populations	Among species	1	37.27	0.5	5	Φ_RT_ = 0.053	0.001
	Among populations within species	13	163.11	0.63	7	Φ_PR_ = 0.070	0.001
	Within populations	87	730.46	8.39	88	Φ_PT_ = 0.119	0.001
	Total	101	930.83	9.53			
Parapatric populations	Among species	1	399.26	10.93	52	Φ_RT_ = 0.524	0.001
	Among populations within species	6	104.18	1.04	5	Φ_PR_ = 0.105	0.003
	Within populations	62	550.16	8.87	43	Φ_PT_ = 0.574	0.001
	Total	69	1053.60	20.84			

df, degree of freedom; SS, sum of squares.

**Figure 4:**
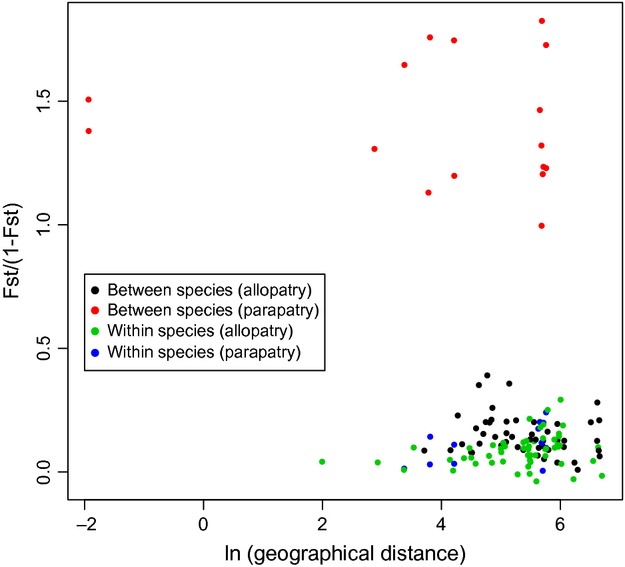
Comparison of isolation by geographical distance between populations. Black and red dots indicate interspecific comparisons between allopatric populations and parapatric populations, respectively, whereas green and blue dots indicate intraspecific comparisons between allopatric populations and parapatric populations, respectively.

**Figure 5:**
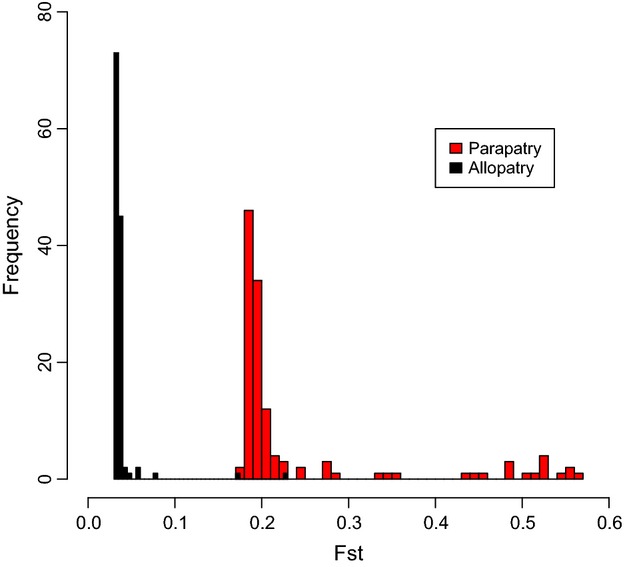
Frequency distributions of locus-specific *F*_ST_ values for comparisons between allopatric (black) and parapatric (red) pairs of populations. The *F*_ST_ distribution tended to be “L-shaped” for allopatric population pairs and shifted to the right for parapatric populations.

Despite the strong population structure observed in both *A*. *chensiensis* and *A*. *fargesii*, there was no evidence for isolation by distance (Mantel test: *r *=* *−0.022, *P *=* *0.499, and *r *=* *−0.069, *P *=* *0.514, for allopatric and parapatric populations, respectively). The lack of evidence for IBD in both allopatric and parapatric populations of each species (Fig. 4) indicates that the strong genetic differentiation between parapatric populations is likely not caused by neutral demographic processes associated with range expansion. Moreover, an examination of the distribution of locus-specific *F*_ST_ values between allopatric and parapatric populations of both species showed that it was strongly “L-shaped”, with most loci showing little or no divergence between allopatric populations (Fig. 5). In contrast, *F*_ST_ values for adjacent parapatric populations were shifted to the right of this distribution (Fig. 5). In line with this, parapatric populations exhibited significantly higher average values of *F*_ST_ relative to allopatric populations (Wilcoxon rank sum test, *P *<* *0.001, mean *F*_ST_ = 0.038 for allopatric populations and *F*_ST_ = 0.240 for parapatric populations). Thus, rather than being restricted solely to outlier loci, it appears that interspecific divergence in parapatry is characterized by widespread genetic differentiation across the genome.

## Discussion

In contrast to an initial expectation that genetic divergence between parapatric populations of two species of *Abies*, *A*. *chensiensis* and *A*. *fargesii* might be reduced due to introgression, we found that divergence based on amplified fragment length polymorphism was much greater between parapatric populations than between allopatric populations. This difference could result from demographic processes associated with range expansions of the two species (Freedman et al. 2010). For example, if parapatric populations of both species were derived from allopatric ones, repeated bottleneck events associated with recent expansion might have lead to increased genetic divergence between parapatric populations due to founder events and genetic drift. However, this hypothesis is inconsistent with our previous phylogeographical study of these two species (Wang et al. 2011). Based on an analysis of cpDNA and mtDNA sequence variation, we previously obtained no evidence that allopatric populations of either species had undergone range expansion to give rise to parapatric populations at least since the penultimate glacial period (Wang et al. 2011). Furthermore, strong genetic drift associated with demographic expansion would result in reduced genetic diversity, which was not evident from comparisons of expected heterozygosity made between allopatric and parapatric populations of either species (Table 1). Finally, no geographical pattern of isolation by distance (IBD) was found for either intraspecific or interspecific comparisons in allopatry and parapatry (Fig. 4), suggesting that geographical distance has played either no role or a minor role in restricting gene flow within and between these species. Because there are no physical barriers to gene flow between allopatric and parapatric populations of both species, the striking pattern of increased divergence between species in parapatry, and also between parapatric and allopatric populations of the same species, cannot result from geographical barriers to gene flow. It would seem, therefore, that neutral genetic drift or other demographic processes are unlikely to have caused the increased genetic divergence observed between the two species in parapatry, although further investigations are needed to rule out these possibilities entirely.

Based on our results, it seems more likely that increased genetic divergence between *A*. *chensiensis* and *A*. *fargesii* in parapatry may reflect the effects of divergent selection (Barton and Bengtsson 1986; Beaumont and Balding 2004; Dayan and Simberloff 2005; Storz 2005; Stinchcombe and Hoekstra 2007; Nosil et al. 2008, 2009; Pfennig and Pfennig 2009) as follows. First, it is feasible that selection may have acted to increase ecological adaptation to different habitats, so as to reduce interspecific competition in areas of species overlap (Schluter 2001; Nosil 2012). This prediction is consistent with field observations showing that while both species grow over the same range of altitude in allopatry, in parapatry, *A*. *chensiensis* occurs mainly at low altitudes while *A*. *fargesii* is found above 2200 m (Guan 1982; Zhang 2001). However, further investigation will be required to prove that adaptive ecological divergence has taken place, possibly involving transplant studies and the analysis of candidate genes responsible for local adaptation at parapatric sites. Second, given that hybridization between recently diverged conifer species is often recorded in the wild, we cannot rule out that selection may have acted to strengthen prezygotic isolation in parapatry (reinforcement) (Noor 1999; Servedio and Noor 2003; Servedio 2004; Petit and Hampe 2006). We have observed occasional individuals in the field exhibiting intermediate morphology to *A*. *chensiensis* and *A. fargesii*, especially in areas of parapatry, indicating that hybridization occurs between these two species. If hybrids were formed frequently in the past and had low fitness relative to parental species, selection for reinforcement might have occurred at parapatric sites causing barriers to gene flow to strengthen (Hopkins 2013). Third, rather than being driven by interspecific interactions in parapatry, local adaptation to biotic and/or abiotic conditions existing between allopatric and parapatric sites occupied by the same species could have indirectly caused the increased genetic divergence between parapatric populations (Nosil 2012; Hopkins 2013). Although we are currently unable to discriminate among these potential selective mechanisms, future studies combining morphological, ecological, and genomic data will hopefully do so and reveal why there is increased genomic divergence between parapatric populations of *A. chensiensis* and *A. fargesii*.

The very low level of AFLP divergence recorded between species based on comparisons between allopatric populations was somewhat surprising given their morphological divergence in allopatry (Liu 1971; Farjon and Rushforth 1989). This suggests that taxonomically important morphological differences between these species might be controlled by relatively few loci and that allopatric populations are genetically similar at most loci (Fig. 5). This prediction is consistent with our previous study, which indicated that the species pair is probably at an initial stage of divergence (Wang et al. 2011). In contrast, higher levels of between species differentiation in parapatry imply that divergent selection might have reduced effective gene flow sufficiently for widespread divergence to accumulate between populations across the genome, instead of being restricted to sites tightly linked to a few loci subject to divergent selection (Feder et al. 2012; Flaxman et al. 2013). Because the distribution of selected sites can vary across the genome, a large degree of heterogeneity in levels of genetic differentiation between parapatric populations might be generated as observed in our study (Fig. 5). This pattern is consistent with one expected under “genome hitchhiking” (Feder et al. 2012). Future comparisons of the genome sequences of these two *Abies* species in parapatry and allopatry are now required to establish the exact level and pattern of genomic divergence, which exists between them.
